# Comparative genomics reveals adaptive traits in novel Antarctic lithic cyanobacteria

**DOI:** 10.1186/s12864-025-12203-7

**Published:** 2025-11-05

**Authors:** Marc W. Van Goethem, Surendra Vikram, Don A. Cowan, Thulani P. Makhalanyane

**Affiliations:** 1 Department of Biochemistry, Centre for Microbial Ecology and Genomics, Genetics and Microbiology, University of Pretoria, Lynnwood Road, Pretoria, 0028 South Africa; 2 Biological and Environmental Sciences and Engineering Division, King Abdullah University of Science and Technology (KAUST), Thuwal, 23955-6900 Kingdom of Saudi Arabia; 3 Department of Microbiology, Faculty of Science, Stellenbosch University, Stellenbosch, 7600 South Africa; 4 The School for Data Science and Computational Thinking, Stellenbosch University, Stellenbosch, 7600 South Africa

**Keywords:** Cyanobacteria, Antarctica, Genome sequencing, Hypolith, Endolith

## Abstract

**Background:**

Terrestrial polar cyanobacteria persist at the cold limits of life, enduring a suite of extreme stressors including sub-zero temperatures, frequent freeze–thaw cycles, oligotrophic soils, variable light regimes with long periods of darkness, and desiccation. To survive, cyanobacteria have evolved diverse physiological strategies. A key adaptation among Antarctic terrestrial cyanobacteria is niche colonization: inhabiting the undersides of translucent quartz rocks (hypoliths) and the interior spaces of porous rocks (endoliths), which buffer environmental extremes and sustains the potential for photoautotrophic carbon fixation. However, the full genomic repertoire facilitating their resilience is incomplete.

**Results:**

We cultivated cyanobacteria from endolithic and hypolithic niches in Victoria Valley, Eastern Antarctica, and recovered four near-complete genomes (100% completeness, < 1% contamination). Three hypolithic genomes showed near-identical sequence similarity (whole genome average nucleotide identity = 99.98%) and phylogenomic proximity to the genus *Coleofasciculus*, yet represent a novel species, *Coleofasciculus caryii* H7-2. The fourth genome, derived from an endolith, showed moderate similarity to *Aliterella antarctica* (whole genome average nucleotide identity = 79.1%), and is proposed as a new species, *Aliterella bergstromii* E5.1.

*C. caryii* H7-2 possessed a larger genome (~ 6.1 Mbp) than *A. bergstromii* E5.1 (~ 5.4 Mbp). Both genomes encoded complete pathways for carbon fixation via oxygenic photosynthesis (RuBisCO and phosphoribulokinase), extensive phycobilisomes, and multiple photoprotective mechanisms. Predicted optimal growth temperatures were 21.7 °C and 23.2 °C, respectively. Shared stress-mitigation genes included those for osmotic, thermal and oxidative (superoxide dismutase) stress response. All genomes contained biosynthetic gene clusters associated with stress-adaptive secondary metabolites, including heterocyst glycolipids, siderophores, phenazines, compounds related to nostopeptolide and merocyclophane. The *C. caryii* H7-2 genome encoded multiple CRISPR-Cas systems, suggesting adaptive immunity and historical phage exposure. In contrast, *A. bergstromii* E5.1 harboured a single prophage and an array of 24 plasmids.

**Conclusions:**

These finding reveal that the newly-described cyanobacteria possess a rich genomic repertoire of adaptations to withstand Antarctic extremes, emphasizing the resilience and ecological importance of lithobiontic cyanobacteria in polar deserts.

**Supplementary Information:**

The online version contains supplementary material available at 10.1186/s12864-025-12203-7.

## Background

Cyanobacteria demonstrate remarkable resilience in some of the most extreme environments on Earth, including rock-associated niches of the hyperarid McMurdo Dry Valleys of Antarctica [1]. As an ancient phylum capable of oxygenic photosynthesis, cyanobacteria are recognised as crucial ‘ecosystem engineers’ in soils of both hot and cold deserts [2]. Cyanobacteria are frequently the primary colonists of lithic niches such as sandstone and quartz rocks [3] embedded within the desert pavement [4]. This strategy protects cells from intense ultra-violet (UV) radiation exposure, and mitigates temperature fluctuations and wind abrasion [5] while increasing water bioavailability [6]. In addition to employing stress avoidance and dormancy strategies, Cyanobacteria harbour diverse metabolic and physiological adaptions to contend with severe, persistent abiotic stressors. For instance, exopolysaccharide (EPS) production facilitates desiccation tolerance, likely by stabilising enzymes and increasing water retention within the cell [7]. Additionally, repair mechanisms against UV-induced DNA damage appear to maintain cellular viability [8].

Some cyanobacteria are able to fix both atmospheric carbon dioxide and nitrogen gases, the former contributing to the oxygenation of Earth’s atmosphere for approximately 2.4 billion years [9]. In desert environments, Cyanobacteria frequently serve as key primary producers and indirectly support heterotrophs by releasing photosynthate into the environment [5]. Metabolic exchange with proximal heterotrophs [10] can create a reciprocal relationship that benefits the cyanobacterium [11]. Understanding these relationships has been central to studying how microbial community ecology is governed in both hypoliths—communities underneath rocks—and endoliths—communities within rocks [1].

While many of the physiological attributes contributing to the success of cyanobacterial in extreme environments have been well-documented [4], the genomic underpinnings of these adaptions are less apparent. Cyanobacterial genomes have been reported from Antarctica, such as the *Phormidesmis priestleyi* isolate from a freshwater lake in the Larsemann Hills [12], while a metagenome-assembled genome (MAG) led to the discovery of a new genus, *Aurora*, that was reconstructed from sequence data obtained from Lake Vanda in the McMurdo Dry Valleys [13]. Here we report the genome sequences of two novel cyanobacterial species isolated from hypoliths and endoliths in Victoria Valley, Eastern Antarctica. These genomes provide new insights into the ecological roles of terrestrial Antarctic cyanobacteria and elucidate the genetic mechanisms that enable their survival in these extreme desert systems.

## Methods

### Sample collection

Sandstone endoliths and quartz hypoliths were collected from Victoria Valley, Antarctica (77°20′ S, 161°39′ E) in January 2013. Collected rock samples were placed in sterile sealed Whirlpak bags for transport at below freezing in the field and during transport to the laboratory (University of Pretoria (UP), South Africa). Cyanobacterial biomass was aseptically isolated from the lithic substrates (hypoliths and endoliths) and was used to cultivate the cells studied here.

### Growth conditions and genomic DNA preparation

Cyanobacterial cultures were grown aerobically on Blue-Green agar (BG-11) at room temperature (~ 21ºC) for two years. Throughout this time a series of subcultures were produced from single cells to enable the axenic propagation of cyanobacteria. Pure isolates were transferred to liquid BG-11 media after individual cells were obtained from the solid media. Isolates were then grown aerobically in liquid BG-11 media, after which cells were collected for DNA isolation. Genomic DNA was isolated using an established phenol–chloroform protocol with final elution volumes of 50 µl [14]. The quantity and quality of the genomic DNA was measured using a NanoDrop™ 2000 Spectrophotometer (Thermo Scientific, Waltham, MA, USA) and was visualised by agarose gel electrophoresis.

### DNA sequencing

DNA isolations from pure cultures with the highest DNA purity and concentration were selected for whole-genome sequencing. The DNA samples (*n* = 4) were sequenced at the Leeds Institute for Molecular Medicine (University of Leeds, UK) on a MiSeq instrument (Illumina®) generating 2 × 250 bp paired-end libraries. The raw paired-end sequences were quality trimmed at a Phred quality score ≥ 20. All reads with ambiguous bases (internal *N*’s) were discarded using Prinseq-lite v0.20.4 [15]. Taxonomic profiling of reads was quantified using the sensitive and highly-specific classification algorithm GOTTCHA2 [16].

### Bioinformatic analysis

Retained high-quality read pairs were individually assembled using SPAdes v.3.7.1 with the –*careful* and –isolate flags implemented with *k*-mer step increases from 21 to 127 [17]. The final assembled scaffolds were then binned into genomes using MetaBAT 2 v1.7 under default parameters with the –*verysensitive* flag used [18]. This relied on metagenomic mapping data in the form of a coverage file to enhance genome binning. Filtered reads were mapped back to their corresponding contigs using bbmap v39.01 [19] and their binary alignment map files were summarized into a depth matrix. CheckM2 v1.0.2 was used to evaluate genome quality and contamination [20]. MAGpurify v2.1.2 was used for further genome refinement by identifying and removing known-contaminant sequences, and removing contigs with outlier tetranucleotide frequencies or outlier G + C content [21]. Genome quality was determined using the current MiMAG standards which include > 50% completeness and < 10% contamination cutoffs, as well as the presence of at least 18 unique tRNAs, and the full ribosomal RNA operon (5S rRNA gene, 16S rRNA gene, and 23S rRNA gene) [22].

Genomes were annotated on the RAST [23] and KAAS servers [24], and using Prokka v1.14.5 [25] within KBase [26]. Final metabolic reconstructions of each genome were distilled with DRAM [27]. Phylogenomic placement of the genomes were inferred using GTDB-Tk v2.4.0 [28] against the latest Genome Taxonomy Database of known genomes (GTDB R226). We used Tome (Temperature optima for microorganisms and enzymes) v1.0 [29] to predict optimal growth temperatures (°C) for each genome from their predicted proteome by training a machine learning model. Next, we used this information to estimate maximal growth rates with gRodon2 [30] by including additional phenotypic trait data [31].

Viral contigs within the bacterial genomes were identified using VirSorter 1.0.5 [32]. Secondary metabolic gene clusters were identified using antiSMASH v8.0.1 under *strict* detection settings and only biosynthetic gene clusters (BGCs) > 10 kb retained for analysis [33]. Antibiotic resistance genes (ARGs) were identified using the Comprehensive Antibiotic Resistance Database (CARD) with the resistance gene identifier (RGI) tool, the results of which were corroborated using our noradab server [34].

We made use of the JSpeciesWS web server [35] for genome-genome comparisons with closely-related species; *Aliterella atlantica* (GCA_000952155.1) and *Coleofasciculus* sp. FACHB-T130 (JACJOG010000001.1). POCP-nf v2.3.6 was used to calculate the percentage of conserved proteins (POCP) among genomes [36]. Proksee was used for circular genome visualization and blast comparisons to the reference genomes [37]. The Genome-to-Genome Distance Calculator (GGDC) was used for digital DNA-DNA hybridization between genomes [38]. Visualization of genome alignments with the closest strains were computed with D-GENIES [39]. We used geNomad v1.8.1 [40] to detect more viruses as well as plasmids within each genome. Plasmids were explored for their conjugation potential using ConJScan model [41] within MacSyFinder v2.1.4 [42]. CRISPR-Cas arrays were detected using minCED v0.4.2 [43].

### Phylogenomic inference

A phylogenomic tree was built using GToTree v1.6.12 [44] to include NCBI accessions listed among representatives of the cyanobacterial phylum, which included 1,067 genome entries from GTDB R226. We used the Cyanobacteria-specific single copy gene (SCG)-set Hidden Markov Models (HMMs) which includes 251 genes to build a multiple sequence alignment. The maximum likelihood tree was built using FastTree v2.1.10 [45] with the Jones-Taylor-Thornton substitution model. Final tree visualization was performed using iTOL v6.5.2 [46]. We made pangenomic comparisons to 21 publicly available cyanobacterial genomes to identify functional gene content differences between our novel genomes and those isolated from other environments. Visualization of the selected genomes was performed using anvi’o v7 [47] under the following parameters: -minbit 0.5 and -mcl-inflation 10. Gene clustering based on amino acid sequence similarity was done by applying a Markov Cluster Algorithm (MCL) thereby grouping genes into homologous clusters based on sequence similarity. For each genome, gene clusters were identified, and their presence or absence was determined across genomes. Anvi’o has the dependencies Prodigal v2.6.3 [48] for gene prediction and MUSCLE v3.8.1551 [49] for multiple sequence alignment.

### Read recruitment analysis

To determine the prevalence of the novel genomes in existing Antarctic microbiome datasets we mapped filtered metagenomic reads to each genome using bbmap v39.01 [19]. Here we relied on our publicly available metagenomes from Antarctic soil [34] and hypolith [50] samples.

## Results and discussion

### Genomic properties

**Fig. 1:**
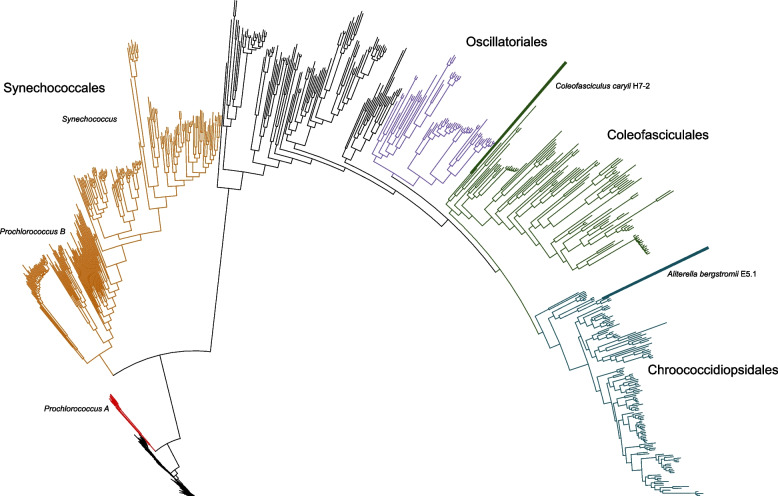
Phylogenomic tree generated with 897 representative cyanobacterial genomes included from the latest Genome Taxonomy Database (GTDB R226). Branches are coloured according to their class. The two strains described here are indicated by text and label bars

The genome properties of the four Antarctic cyanobacterial isolates are summarized in Table 1. The three hypolithic genomes shared exceptionally high whole-genome average nucleotide identity (ANI; 99.98%), and we selected the H7-2 genome for further investigation due to its high completeness (100%), low contamination (0.39%) and the fewest contigs among the three isolates (*n* = 116 contigs). Phylogenomic analysis based on 49 core, universal bacterial genes revealed that the H7-2 genome clusters closely with the terrestrial cyanobacterial isolate *Coleofasciculus* sp. FACHB-T130 (GCF_014695375.1 [51]; Fig. 1).

**Table 1:** Genomic features of the four novel cyanobacterial genomes; *Aliterella bergstromii* E5.1, *Coleofasciculus caryii* H7-2, *C. caryii* H7-1, and *C. caryii* H7-3

Genome	Completeness (%)	Contamination (%)	Genome Size (bp)	Coding Density	Contig *N50*	Average Gene Length (bp)	G + C Content (%)	Total Coding Sequences	Total Contigs	Max Contig Length (bp)	No. of tRNAs	5S rRNA	16S rRNA	23S rRNA	MiMAG classification	GTDB-Tk classification against GTDB R226	GTDB-Tk RED Score	predOGT (°C)	Minimal doubling time (hrs)
*Aliterella bergstromii* E5.1	100	0.91	5,352,456	0.843	36,813	260.5	42.97	5814	533	186,126	50	1	1	1	Near-complete	d__Bacteria;p__Cyanobacteriota;c__Cyanobacteriia;o__Cyanobacteriales;f__Chroococcidiopsidaceae;g__Aliterella;s__	0.95955	23.26	19.01
*Coleofasciculus caryii* H7-2	100	0.39	6,167,052	0.818	137,907	305.8	46.79	5507	116	329,368	75	4	2	2	Near-complete	d__Bacteria;p__Cyanobacteriota;c__Cyanobacteriia;o__Cyanobacteriales;f__FACHB-T130;g__FACHB-T130;s__	0.96835	21.70	14.29
*Coleofasciculus caryii* H7-1	85.98	0.71	5,461,250	0.823	122,095	309.5	46.71	4847	88	399,679	70	2	1	1	Medium-quality draft	d__Bacteria;p__Cyanobacteriota;c__Cyanobacteriia;o__Cyanobacteriales;f__FACHB-T130;g__FACHB-T130;s__	0.97185	23.24	18.70
*Coleofasciculus caryii* H7-3	100	1.29	6,241,337	0.821	611,174	304.7	46.82	5616	284	254,334	79	1	1	2	Near-complete	d__Bacteria;p__Cyanobacteriota;c__Cyanobacteriia;o__Cyanobacteriales;f__FACHB-T130;g__FACHB-T130;s__	0.97286	23.23	16.68

The H7-2 genome had a G + C content of 46.79%, which is marginally higher than *Coleofasciculus* sp. FACHB-T130 (46.7%), while the genome size of H7-2 was slightly smaller (6.17 Mbp *vs*. 6.21 Mbp). Despite sharing 90.8% whole genome average nucleotide identity (ANI) with *C.* sp. FACHB-T130 (Supplementary Table S1), the highest 16S rRNA gene similarity of H7-2 was observed with *Funiculus sociatus* SIK29 (98% identity). Further genomic comparisons with *Coleofasciculus* sp. FACHB-T130 for species-level delineation indicated similarity scores below species-level thresholds (including ANIb ≥ 95%, ANIm ≥ 95%, tetranucleotide correlation searches ≥ 0.999, dDDH ≥ 70%) and > 50% percentage of conserved proteins (POCP) for genus level delineation (Supplementary Figures S1 and S2). Based on these findings, we propose the designation of a new species within the genus *Coleofasciculus*. Comparative genomic analysis revealed marked differences in genome content, supporting the classification of H7.2 as a novel cyanobacterial strain. In recognition of its phylogenomic distinctiveness, we propose the name *Coleofasciculus caryii* H7-2, recognising Craig Cary for his substantial contribution to Dry Valley soil microbiology.

**Fig. 2:**
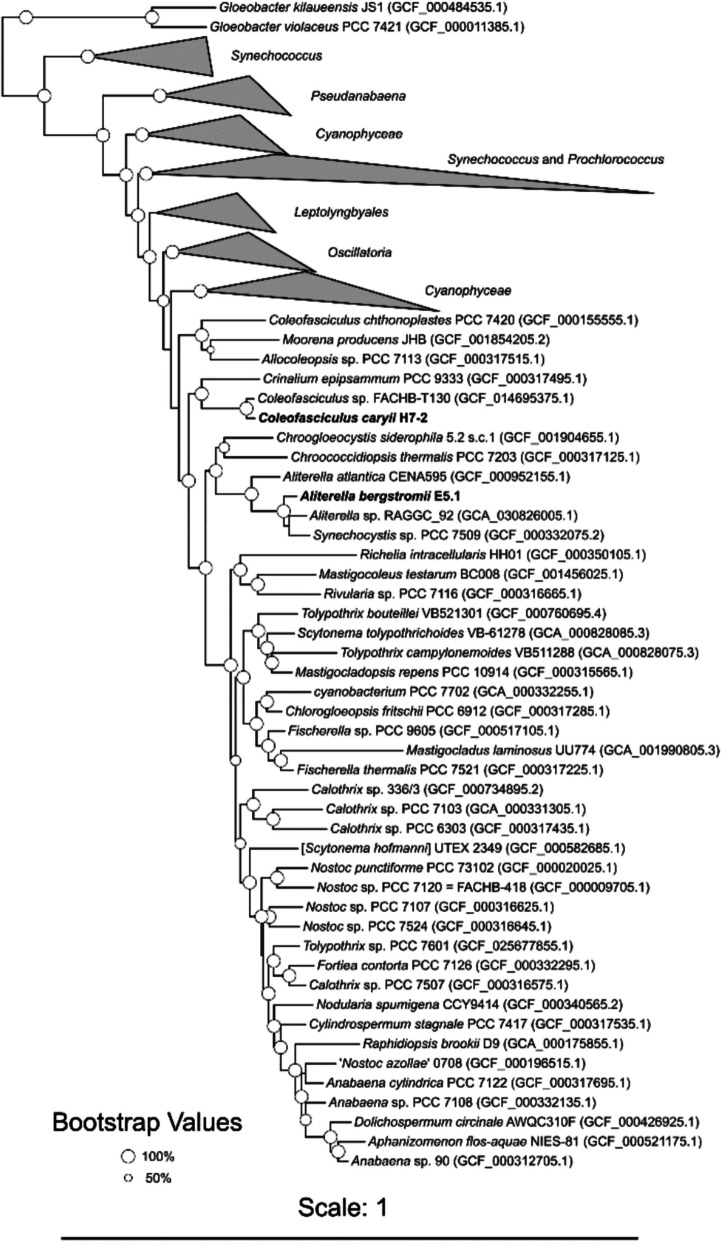
Maximum likelihood tree with 200 representative RefSeq genomes included for delineation of closely-related genomes. Bootstrap values are indicated as circles that are proportional to their support, i.e. larger circles indicate higher bootstrap support. *Gloeobacter* reference genomes were selected as the outgroup as a sister group to all other cyanobacteria

The endolith E5.1 genome, 100% completeness and 0.91% contamination, showed the highest similarity to *Aliterella atlantica* CENA595 (GCF_000952155.1) and clustered phylogenomically among members of the *Aliterella* genus (Fig. 2) [52]. The reference strain *A. atlantica* CENA595 was isolated from deep waters on the South Atlantic Ocean continental shelf [52]. Other species in this genus have been discovered across a variety of habitats, including *A. chasmolithica* in granitic stones from the arid Atacama Desert, Chile [53], *A. shaanxiensis* from a freshwater lake in China [54], and *A. vladivostokensis* from an urban environment in Vladivostok, Russia [55].

E5.1 is comparable to *A. atlantica* both in terms of G + C content (42.98% *vs.* 42.6%, respectively) and genome size (5.38 Mbp *vs*. 5.27 Mbp; Table 2). The two genomes shared 79.3% ANI (Supplementary Table S1) and 96% 16S rRNA gene similarity. Further genomic comparisons with *Aliterella atlantica* CENA595 and *Aliterella* sp. RAGGC 92 (GCA_030826005.1) for species-level delineation provided similarity scores below the species-level thresholds for both genomes (including ANIb, ANIm, tetranucleotide correlation searches, dDDH and POCP and Supplementary Figures S3 and S4). Based on these genomic distinctions, we propose the designation of a new species within the *Aliterella* genus. Given its abundance of plasmids, which likely facilitate gene sharing, and its phylogenomic novelty (see Results section), we propose the name *Aliterella bergstromii* E5.1, recognising Dana Bergstrom for her significant contributions to Antarctic research and science communication.

**Table 2:** Biosynthetic Gene Clusters (BGCs) longer than 10 kb recovered from the cyanobacterial genomes *Aliterella bergstromii* E5.1 and *Coleofasciculus caryii* H7-2

Sample	BGC Type	Most similar known cluster	Similarity	Size (bp)	Full-length cluster
*Aliterella bergstromii* E5.1	T1PKS	Merocyclophane C/D	66%	28,787	No
*Aliterella bergstromii* E5.1	NRPS	microginin	28%	22,042	No
*Aliterella bergstromii* E5.1	Terpene	Capsular polysaccharide	13%	20,926	Full-length
*Aliterella bergstromii* E5.1	Siderophore	schizokinen	100%	25,023	No
*Aliterella bergstromii* E5.1	Phenazine			13,647	No
*Aliterella bergstromii* E5.1	mycosporine-like amino acids	hexose-palythine-serine/hexose-shinorine	42%	48,124	Full-length
*Coleofasciculus caryii* H7-2	NRP-metallophore;NRPS;T1PKS	anachelin	35%	57,170	No
*Coleofasciculus caryii* H7-2	NRPS-T1PKS	Malyngamide I	12%	50,021	No
*Coleofasciculus caryii* H7-2	Terpene			20,929	Full-length
*Coleofasciculus caryii* H7-2	hglE-KS	Heterocyst glycolipid	57%	54,141	Full-length
*Coleofasciculus caryii* H7-2	NRPS	microginin	42%	57,103	Full-length

### Carbon dioxide fixation, photosynthesis and light harvesting

Both H7-2 and E5.1 genomes contained complete photosystem II (PSII) reaction centre II genes (*psbAD*) and nearly complete Calvin-Benson-Bassham cycles, with 10 of the 11 core genes, including key carbon fixation genes ribulose bisphosphate carboxylase/oxygenase large- and small-subunit genes (*cbbLS*) and phosphoribulose (*prk*). Both genomes also possessed numerous key genes for PSII, including *psbBEFLMNTZ* and *psb27*; however the crucial *psbJ* gene is absent from the H7-2 genome. The *psbEFLJ* gene cluster is highly conserved and commonly found in many *Cyanobacteria*, such as *Synechocystis* sp. PCC 6803 [56] as well as in higher plants, where the PsbJ protein plays a critical role in controlling the assembly of functional PSII complexes in the thylakoid membrane.

Although *psbJ* is an intrinsic component of the PSII complex, cyanobacteria lacking this gene can still perform photosynthesis [57], albeit with lower rates of CO_2_ production, and less stable D1/D2 dimers. In the thermophilic cyanobacterium *Thermosynechococcus elongatus*, Δ*psbJ* mutants accumulate Psb27-Psb28 photosystem II complexes that form monomeric PSII units with reduced stability and lower oxygen-evolving capacity [57].

The genomic determinants for phycobilisomes of both novel cyanobacteria were extensive, comprising both allophycocyanin (*apcABCED*) and phycocyanin components (*cpcABCDEFGST*) which mainly absorb red light. However, their phycoerythrin genes were limited, with only *cpeS2* present, which encodes a putative phycocyanobilin lyase. In addition to light-sensitive reactions, both genomes possess the potential for light-independent processes, with genes encoding dark-operative protochlorophyllide reductases (*chlBLMN* and *bchBI*), enabling them to reduce protochlorophyllide without light. The *C. caryii* H7-2 genome encodes a copy of the *pixJ* gene, which encodes a phototaxis photoreceptor of the cyanobacteriochrome family [58]. This light-sensing domain could be involved in phototaxis by adjusting the cells position to directional light to optimize photosynthesis and minimize photodamage under high light conditions [59]. The E5.1 genome had two copies of the chlorophyll-binding protein PcbABC (*isiA*) that was not present in other cyanobacterial genomes (List of unique gene clusters in E5.1 and H7-2 are listed in Supplementary Table S2). These proteins are typically induced by high light or iron-deficient conditions so that IsiA proteins assemble around PSI into super complexes that can dissipate excess excitation energy (quenching), as shown in the marine cyanobacterium *Synechocystis* PCC 6803 [60]. The E5.1 genome, unlike H7-2 and the reference cyanobacterial genomes, has genes with similarity to bacteriorhodopsins, which are light-driven proton pumps with potential roles as light-energy-harvesting systems [61]. Rhodopsins could support certain lineages with near-constant energy supply in the form of light through energy conservation, a particularly beneficial trait in nutrient-poor Antarctic soils where it is suspected that cyanobacterial photoautotrophs can conserve energy when water for oxygenic photosynthesis is limiting [62].

**Fig. 3:**
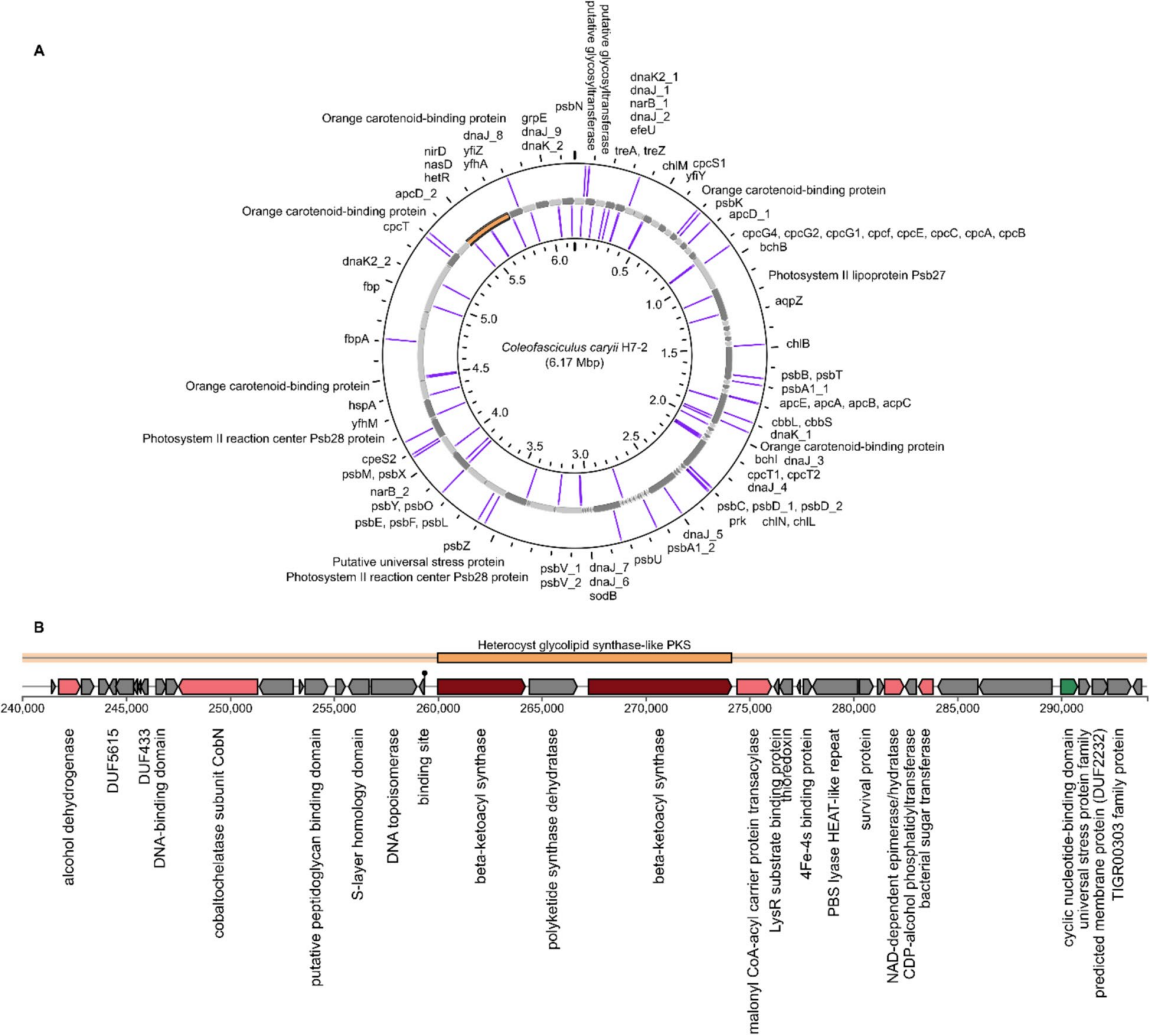
**A** Circular genomic representation of *Coleofasciculus caryii* H7-2 with key genes for stress mitigation, photosynthesis and nitrogen cycling indicated. The contig coloured in orange corresponds to **B**) the heterocyst glycolipid biosynthetic gene cluster region

Both Antarctic genomes, and their reference genomes, encoded enzymes for the breakdown of arabinan, a polysaccharide. Notably, the *C. caryii* H7-2 genome harbours a broader array of carbohydrate-active enzymes (CAZymes) designed for metabolising various polymers (Fig. 3A). These include enzymes targeting amorphous cellulose, xyloglucan (linear polysaccharides), and mixed-linkage glucan (hemicellulosic polysaccharides; Supplementary Table S3). We hypothesize that the breakdown of these complex compounds, some of which are found in EPS matrices [63], may augment carbon uptake, representing a heterotrophic scavenging strategy crucial for cellular survival during the austral winter when sunlight is largely or completely absent for six months.

### Photoprotection

Cellular photoprotection is essential for survival in the Antarctic terrestrial habitats, where the austral summer provides continuous daylight for extended periods, during which, high levels of incident ultra-violet (UV) radiation, particularly UV-B, pose significant risks to cellular components such as DNA and proteins.

**Fig. 4:**
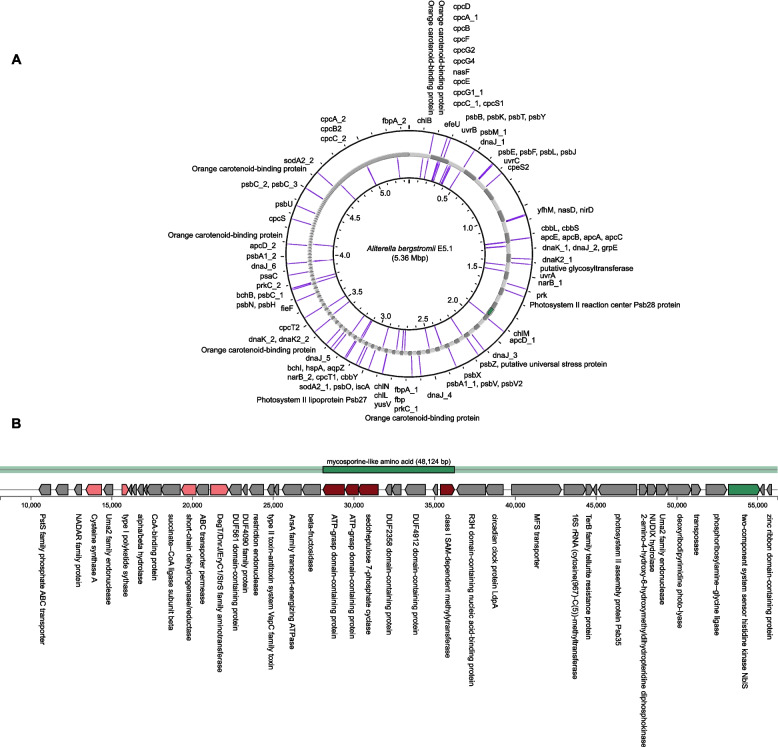
**A** Circular genomic representation of *Aliterella bergstromii* E5.1 with key genes for stress mitigation, photosynthesis and nitrogen cycling indicated. The contig coloured in green corresponds to **B**) the mycosporine-like amino acid biosynthetic gene cluster region

Under intense light conditions Cyanobacteria are susceptible to photoinhibition, where the absorption of photons exceeds the capacity for electron dissipation via photochemical pathways, leading to the accumulation of reactive oxygen species (ROS) at the PSII reaction centre. However the deactivation of PSII is a prominent mitigation mechanism in cyanobacteria [64]. Alternatively, cyanobacteria can employ orange carotenoid proteins (OCPs) which reduce energy transfer from the phycobilisome to PSII and PSI [65]. This mechanism appears critical for cyanobacteria in the Dry Valleys lithic habitats, as the H7-2 genome encodes five copies of OCP gene, while the E5.1 genome encodes six (Fig. 4A). Both cyanobacteria were isolated from colonized lithic communities, where the translucent substrates reflect a significant proportion of incident sunlight, yet the need for robust photoprotective mechanisms appears crucial for these cyanobacteria.

### Genomic adaptations to light-induced damage

In addition to photoprotection mechanisms, both genomes exhibited several putative adaptations to mitigate light-induced oxidative damage, as is common for Cyanobacteria. Both genomes encoded two copies of superoxide dismutase A (*sodA*), an enzyme that protects cells by neutralizing ROS [66], and these were common in all cyanobacterial reference genomes as well. Both genomes carried two copies of rubredoxin (*rubB*) genes, which also play a role in the reduction of superoxides [67] and maintaining PSII activity [68]. Another common stress-response adaptation is alkyl hydroperoxide reductases, enzymes that reduce organic peroxides, including reactive nitrogen intermediates. The *A. bergstromii* E5.1 genome contained six copies of the gene encoding this enzyme, while the H7-2 genome had five. We suggest that these adaptations collectively enhance the ability of cyanobacteria to survive the extreme oxidative stress caused by prolonged exposure to high levels of radiation and desiccation in the Antarctic desert soils and the associated lithic niches.

### Nitrogen metabolism

Antarctic soils are typically nutrient poor and are very low in organic nitrogen content [69]. Acquiring nitrogen from the environment is thus a critical step in maintaining cellular function in this extreme environment. Neither genome contained any genes for nitrogen fixation, indicating non-diazotrophic lifestyles. The H7-2 genome encoded a heterocyst glycolipid, typically associated with *Nostocales* and *Stigonematales*, which use thick cell envelopes to protect the oxygen-sensitive nitrogenase enzyme from the rest of the cell [70]. The presence of a 54,142 bp gene cluster encoding a heterocyst glycolipid in the *C. caryii* H7-2 genome is cryptic, yet was also found in the H7-1 and H7-3 genomes (Fig. 3B). We speculate that heterocyst formation could create a microoxic environment suitable for storing reserve nitrogen under nitrogen-limited conditions, a crucial function in nitrogen management for filamentous cyanobacteria [71]. Additionally, the H7-2 genome also encoded the key heterocyst differentiation control protein, *hetR*, which is autoregulated and activated under nitrogen deficiency. The *hetR* gene in H7-2 shows 78.2% identity to the *hetR* gene in *Microcoleus* sp. PCC7113, while the heterocyst gene cluster shares 82% identity with *Nostoc* sp. NIES-4103 across 43% of the gene cluster.

Both cyanobacteria could influence the nitrogen cycle through denitrification, the reduction of nitrate to nitrite—catalysed by the *narB* gene—and then nitrite to nitric oxide using *nirD* and *nasD*. In the H7-2 genome, nitrite reductase genes were located downstream of *hetR* gene, suggesting potential regulatory coupling between heterocyst differentiation and nitrogen metabolism. Both the heterocyst glycolipid gene cluster and the *hetR* gene are also present in the *Coleofasciculus* sp. FACHB-T130 reference genome.

### Biosynthetic gene clusters (BGCs)

The *C. caryii* H7-2 genome contains five biosynthetic gene clusters (BGCs; Table 3), including non-ribosomal peptide synthetase (NRPS) clusters with similarity to those driving the synthesis of anachelin, malyngamide I, and microginin, all of which have potential cytotoxic properties [72]. The *A. bergstromii* E5.1 genome harbours six BGCs larger than 10 kb (Table 3), including clusters with sequence similarity to known clusters for merocyclophane, microginin, and a capsular polysaccharide. *A. bergstromii* E5.1 also contains genes that encode a siderophore with similarity to schizokinen and a truncated phenazine. The siderophore gene cluster spans 25,023 bp and is likely involved in iron (Fe) chelation from the environment, a critical co-factor in photosynthesis that is often limited in availability [73, 74]. The gene cluster is a full-length mycosporine-like amino acid (MAA) that is encoded a gene cluster that is 48,124 bp long (Fig. 4B) and has 42% sequence similarity to a shinorine MAA identified in *Scytonema* cf. *crispum* [75]. MAAs have putative functions such as protection against radiation by absorbing both UV-A and UV-B radiation in cyanobacteria and releasing UV radiation as heat [76].

**Table 3:** Genome sizes (bp) of the four novel cyanobacterial genomes compared with 19 cyanobacterial reference genomes

Genome	Genome Size (bp)	Accession
*Aliterella bergstromii* E5.1	5,352,456	This study
*Coleofasciculus caryii* H7-2	6,167,052	This study
*Coleofasciculus caryii* H7-1	5,461,250	This study
*Coleofasciculus caryii* H7-3	6,241,337	This study
*Pseudanabaena cinerea* FACHB-1277	4,772,465	GCF_14696345.1
*Calothrix anomala* FACHB-343	9,242,718	GCF_14696435.1
*Calothrix parietina* FACHB-288	9,242,072	GCF_14696555.1
*Nostoc parmelioides* FACHB-3921	7,774,718	GCF_14696625.1
*Anabaena sphaerica* FACHB-251	6,198,606	GCF_14696825.1
*Anabaena subtropica* FACHB-260	5,809,825	GCF_14697105.1
*Anabaena lutea* FACHB-196	6,059,416	GCF_14698305.1
*Microcystis viridis* FACHB-1342	4,697,269	GCF_14698335.1
*Microcystis flos-aquae* FACHB-1344	5,257,036	GCF_14698375.1
*Nostoc spongiaeforme* FACHB-130	7,162,047	GCF_14698475.1
*Nostoc foliaceum* FACHB-393	8,870,024	GCF_14698505.1
*Anabaena catenula* FACHB-362	6,234,385	GCF_14698735.1
*Aphanizomenon flos-aquae* FACHB-1287	4,347,270	GCF_14698755.1
*Nostoc paludosum* FACHB-159	9,270,090	GCF_14698835.1
*Crinalium* SMAG_U16487	8,429,677	DATNPS010000031.1
*Crinalium* SMAG_U16486	2,670,601	DATNPR010000605.1
*Aliterella* RAGGC_92	4,172,295	JAPJOW010000060.1
*Allocoleopsis franciscana* PCC7113	7,966,510	GCA_000317495.1
*Coleofasciculus* FACHB-T130	6,211,392	JACJOG010000001.1

Along with its siderophore, the E5.1 genome encodes several genes potentially linked to iron import, including a putative siderophore transport system ATP-binding protein (*yusV*), ferrous iron permease (*efeU*), iron-binding protein (*iscA*), ferrous-iron efflux pump (*fieF*), and major ferric iron-binding protein (*fbpA*). The genome also contains *psaC* genes for the photosystem I iron-sulfur center and the *chlL* gene for light-independent protochlorophyllide reductase iron-sulfur ATP-binding protein, both essential for efficient photosynthesis under iron-limited conditions. Although H7-2 does not encode a siderophore BGC, it includes four genes associated with the siderophore transport system permease proteins (*yfhA*, *yfhM*, *yfiY*, and *yfiZ*), indicating alternative strategies for iron acquisition.

### Antibiotic resistance genes

Microorganisms have evolved diverse mechanisms to mitigate the toxicity of antibiotics, such as efflux pumps, modification of drug targets, and enzymatic inactivation of antibiotics [77]. In the extreme Antarctic environment, bacteria face intense competition for limited resources [78], often producing and defending against antibiotics [34]. In the genome of *A. bergstromii* E5.1, we identified two key antibiotic resistance genes: TEM-166, a beta-lactamase that likely confers resistance to beta-lactam antibiotics [79], and *adeF*, which encodes a specific efflux pump for fluoroquinolone and tetracyclines. *C. caryii* H7-2 only harboured the *adeF* gene.

### Stress responses

In a likely response to the extreme environmental conditions of Antarctic desert soils, characterized by hyperaridity, hyperoligotrophy and high levels of UV radiation, both cyanobacterial genomes displayed an array of stress response mechanisms. Genes associated with oxidative stress and heat shock were particularly prominent, as revealed by RAST subsystems annotations. Additionally, open reading frames (ORFs) related to DNA repair were prevalent, reflecting the need for constant maintenance of genomic integrity in this hostile environment. Less frequent but still notable were genes involved in osmotic stress responses, sigma B stress response regulation, and cellular detoxification, indicating the multifaceted strategies the cyanobacteria employ to survive in poly-extreme conditions.

In terms of oxidative stress there were numerous genes common to both genomes including: glutathione synthetases, glutathione reductases, glutathione S-transferases, and alkyl hydroperoxide reductase subunit C-like proteins. Unique oxidative traits included catalase and manganese superoxide dismutase (MnSOD) genes in E5.1, and iron superoxide dismutase (FeSOD) and metallothionein genes in H7-2.

### Water scarcity and cellular adaptation

One of the most formidable challenges to life in McMurdo Dry Valley soils is the persistent scarcity of water. In this region, evapotranspiration significantly exceeds precipitation, resulting in a typical soil water content between 0.5—2% (d.w.) [78]. Annual precipitation, delivered exclusively as snow, averages just 10 cm per year [80]. This severe aridity places considerable importance on the mechanisms for water acquisition [81] and internal mobilization within cells. Both cyanobacterial genomes encoded for aquaporin Z (*aqpZ*), an integral membrane protein responsible for the osmotically driven transport of water. Notably, E5.1 contained two copies of the *aqpZ* gene. These water channels play a critical role in cyanobacteria, regulating cell volume, and osmotic stress responses [82]. In *Synechocystis* mutants deficient in *aqpZ*, the inability to regulate cytoplasmic volume under salt stress increased the vulnerability of both PSI and PSII to salt and high-light damage, underscoring the potential importance of AqpZ in PSI and PSII repair following photodamage [83]. In terms of protection from desiccation, we found both trehalose synthase (*treA* and *treZ*), and sucrose synthase genes, suggesting the production of compatible solutes, in both genomes.

Only the E5.1 genome encoded a cold-shock protein (*cspC*), and this gene was not present in any other cyanobacterial genomes analysed (Supplementary Data S2). Both Antarctic genomes showed a presence of stress-inducible heat shock genes, including *dnaJ*, *dnaK* and *grpE*. These heat shock proteins function as molecular chaperones with broad roles in protein homeostasis, including the reactivation of misfolded proteins, disaggregation of protein aggregates, and the transport of proteins across membranes [84]. Their abundance in both genomes suggests that heat shock pathways might compensate for temperature stress in these cyanobacteria, a strategy that could be especially important during fluctuations in environmental conditions, such as freeze–thaw cycles [85].

### Phage infection and mobile genetic elements (MGEs)

**Fig. 5:**
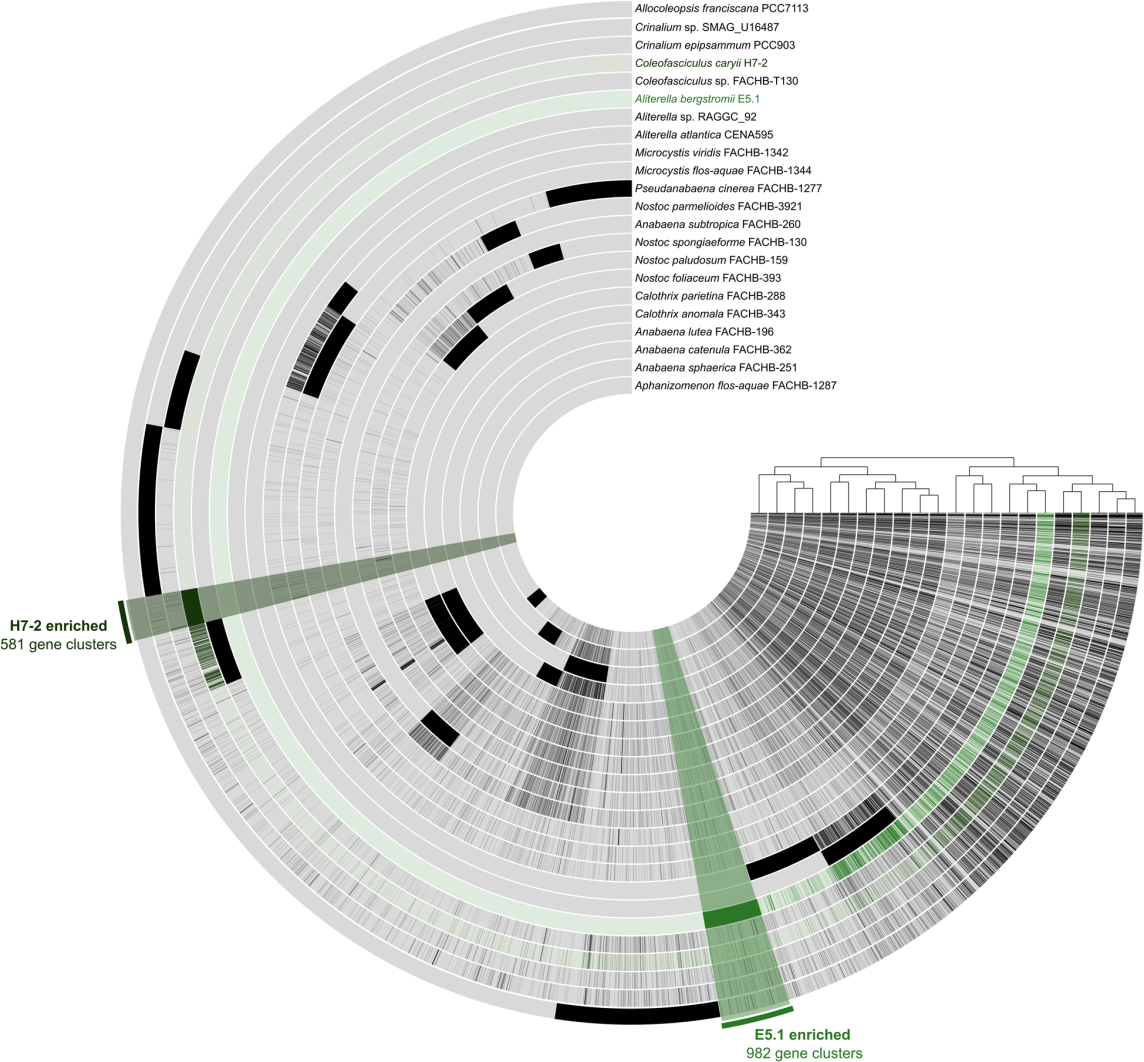
Pan-genome plot displaying gene clusters detected in Antarctic cyanobacterial genomes E5.1 and H7-2 in green, while those detected in the reference genomes are shown in black. Light colours indicate the absence of gene clusters, The E5.1 and H7-2 specific gene clusters are reported in green. Functional annotation on the gene clusters is reported in Supplementary Table S2

Antarctic hypolithic communities are known to host diverse viral populations, predominantly tailed double-stranded DNA (dsDNA) phages from the *Caudoviricetes* order [86]. In response to invader-derived infections, bacterial hosts have evolved various innate and adaptive immune mechanisms to defend against phage predation [87], one of the most prominent being the adaptive CRISPR-Cas system. The *C. caryii* H7-2 genome features a CRISPR-Cas system resembling a Type I-C system (*cas6*-*cas3*-*cas8a1*-*cas7*-*cas5*-*cas4*-*cas1*-*cas2*), suggestive of previous viral encounters. In total the genome harbours four CRISPR arrays containing 679 spacers, of which 674 were unique. Prokaryotic defence using the CRISPR-Cas system was a feature particularly enriched in the H7-2 genome compared to other cyanobacterial genomes with numerous effector subunits and endoribonucleases uniquely associated with the H7-2 genome (Fig. 5, Supplementary Table S2). For example, the *A. bergstromii* E5.1 genome only had two CRISPR-Cas arrays containing 24 spacers, and contained a 12 kb viral genome fragment identified as a dsDNA *Caudoviricetes* phage, potentially a prophage.

Both cyanobacterial genomes exhibited substantial plasmid diversity. We identified 24 potential plasmids within the E5.1 genome, while H7-2 harboured four plasmids, as predicted by geNomad [40]. Consistently, E5.1 had several unique transposases within its genome compared with other cyanobacterial isolates (Fig. 5, Supplementary Table S2). Regardless, none of the plasmids encoded conjugative systems such as T4SS indicating that they are not mobilizable. Plasmid-encoded genes included amylases, various transporters (notably ABC transport domains and sugar transporters), and genes-encoding flavodoxin short-chain proteins that may have roles in stress response such as low iron concentrations, as they can serve as substrates for ferredoxin, an iron-containing protein. The predominant functions of plasmid-located genes were related to DNA integration, transposition, recombination, transmembrane transport, phosphorelay signal transduction, and secondary metabolite biosynthesis, including the production of phenazine and indigoidine, a blue pigment. Phenazines can modulate stress by serving as redox-active compounds that shuttle electrons between intra- and extracellular acceptors thus preventing the build-up of ROS in the cell. Moreover, in iron-limited environments, phenazines have been shown to act as alternative electron acceptors, offering a metabolic advantage during oligotrophy. Indigoidine, a natural biopigment, could serve as a UV protectant, while a role in metal binding is suggestive of reducing cellular toxicity resulting from metal build-up [88].

### Prevalence in Antarctic microbiome datasets

To assess the prevalence of the two novel cyanobacterial species in different Antarctic soil niches we mapped filtered metagenomic reads to calculate the relative proportion of each genome sample. Here we relied on 18 published Antarctic soil metagenomes [34] and a hypolith metagenome [50]. Overall the mapping rates were very low (~ 0.08%) indicating that these species represent only a minor contributions to the community diversity (Supplementary Table S4). However the *A. bergstromii* E5.1 genome was much more common in some samples, reaching 1.39% of the community in a soil sample from Mount Suess, Mackay Glacier region [34], for an average of 0.14% across all metagenomes, while *C. caryii* H7-2 only comprised 0.01% of the metagenomes on average. Both genomes constitute 0.07% of the hypolith metagenome.

## Conclusions

This study presents a comprehensive genomic characterisation of two novel Antarctic cyanobacteria, *Coleofasciculus caryii* H7-2 and *Aliterella bergstromii* E5.1, each representing new species within their respective genera. Through high-resolution phylogenomics and whole-genome comparisons, both isolates were shown to be phylogenetically and functionally distinct from their closest known relatives. Their genomes revealed a repertoire of genomic adaptations enabling survival in the poly-extreme conditions of the Antarctic Dry Valleys. These include genomic determinants for photosynthesis under low light, robust photoprotective strategies, oxidative stress responses, and desiccation resistance.

*Coleofasciculus caryii* H7-2 has a suite of carbohydrate-active enzymes, suggesting a capacity for heterotrophic carbon scavenging, as well as a cryptic heterocyst glycolipid biosynthetic cluster, which may contribute to nitrogen storage or microaerobic niche creation. In contrast, *Aliterella bergstromii* E5.1 is enriched in biosynthetic gene clusters including a full-length mycosporine-like amino acid gene cluster and siderophore pathways, potentially conferring UV and iron-stress tolerance, respectively. Both genomes contain ARGs and CRISPR-Cas systems, indicative of genomic plasticity and phage interactions within their microbiomes.

Altogether, these findings deepen our understanding of the functional capacity and evolutionary trajectories of Antarctic cyanobacteria, shedding light on the genomic basis for persistence and ecological success in one of Earth’s most inhospitable environments. These genomes provide a valuable resource for further exploration of extremophile biology, bioprospecting, and microbial biogeography in polar ecosystems.

## Supplementary Information

Supplementary Material 1.

## Data Availability

The assembled genomes are available at NCBI SRA under the BioProject accession PRJNA1165153. The metagenomes used for read mapping are available at PRJNA376086 and PRJNA175234. The biosynthetic gene clusters (BGCs) are available on Zenodo: *https://zenodo.org/records/15547537.*

## Abbreviations

ANI: Average nucleotide identity
ANIb: Average nucleotide identity through BLAST
ANIm: Average nucleotide identity through Mummer
ARGs: Antibiotic resistance genes
BGCs: Biosynthetic gene clusters
bp: Base pairs
d.w.: Dry weight
dDDH: Digital DNA-DNA Hybridization
dsDNA: Double-stranded DNA
EPS: Exopolysaccharide
MAG: Metagenome-assembled genome
Mbp: Mega base pairs
ORF: Open Reading Frame
POCP: Percentage of conserved proteins
UV: Ultra-violet
