# Supplementary material for: Comparative genomics reveals adaptive traits in novel Antarctic lithic cyanobacteria
Source: BMC Genomics. 2025 Nov 5;26:994. doi: 10.1186/s12864-025-12203-7 (PMC12587747; doi:10.1186/s12864-025-12203-7)
Supplement: Supplementary file 1 — Supplementary Material 1. [file 12864_2025_12203_MOESM1_ESM.docx]

**Supplementary Data for “Comparative genomics reveals adaptive traits in novel Antarctic lithic cyanobacteria”**

Marc W. Van Goethem^1,2^, Surendra Vikram^1^, Don A. Cowan^1^, and Thulani P. Makhalanyane^3,4^

^1^Centre for Microbial Ecology and Genomics, Department of Biochemistry, Genetics and Microbiology, University of Pretoria, Lynnwood Road, Pretoria 0028, South Africa

^2^Biological and Environmental Sciences and Engineering Division, King Abdullah University of Science and Technology (KAUST), Thuwal 23955-6900, Kingdom of Saudi Arabia

^3^Department of Microbiology, Faculty of Science, Stellenbosch University, Stellenbosch 7600, South Africa

^4^The School for Data Science and Computational Thinking, Stellenbosch University, Stellenbosch 7600, South Africa

**Supplementary Figure 1.** Genome comparison between *Coleofasciculus caryii* H7-2 (inner ring, contigs indicated as grey bars) and the closest available reference strain *Coleofasciculus* sp. FACHB-T130 (outer ring, sequence similarities are indicated by red colours).

**
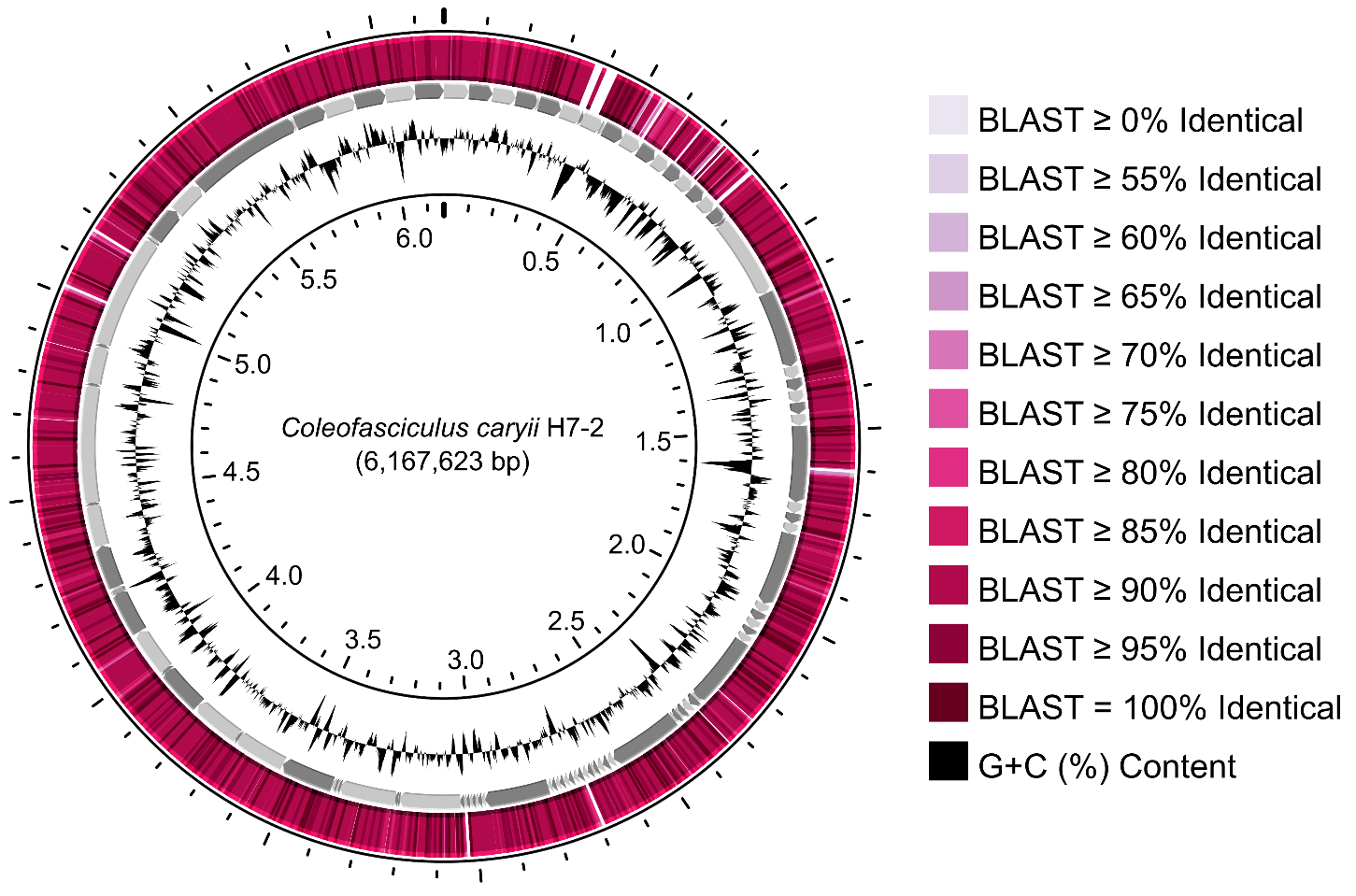
**

**Supplementary Figure 2.** Dot plot showing a genome-wide comparison between *Coleofasciculus caryii* H7-2 and the closest available reference strain *Coleofasciculus* sp. FACHB-T130. The alignments are coloured according to their sequence similarity.

**
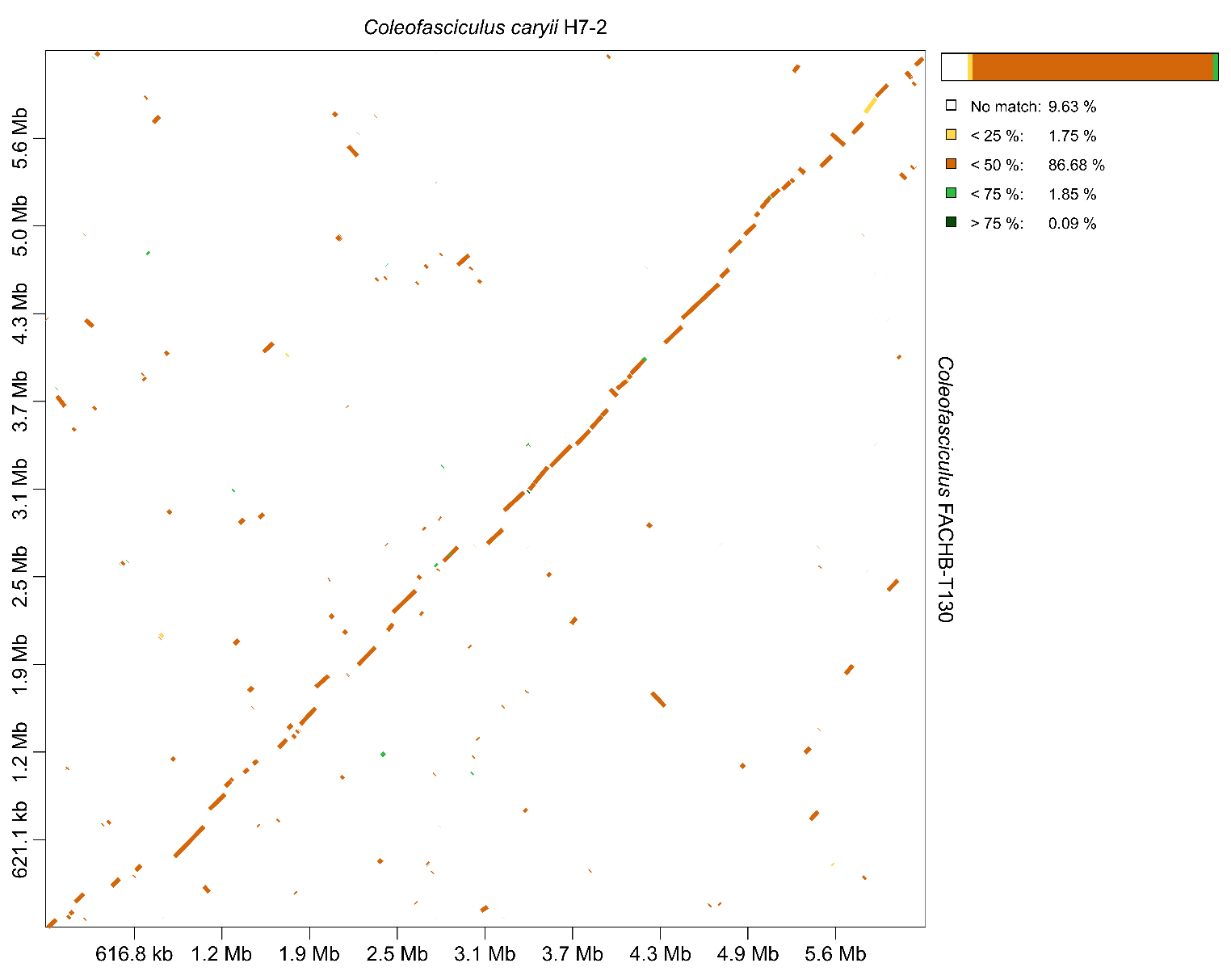
**

**Supplementary Figure 3. Supplementary Figure 1.** Genome comparison between *Aliterella bergstromii* E5.1 (inner ring, contigs indicated as grey bars) and the closest available reference strain *Aliterella* sp. RAGGC 92 (outer ring, sequence similarities are indicated by red colours).

**
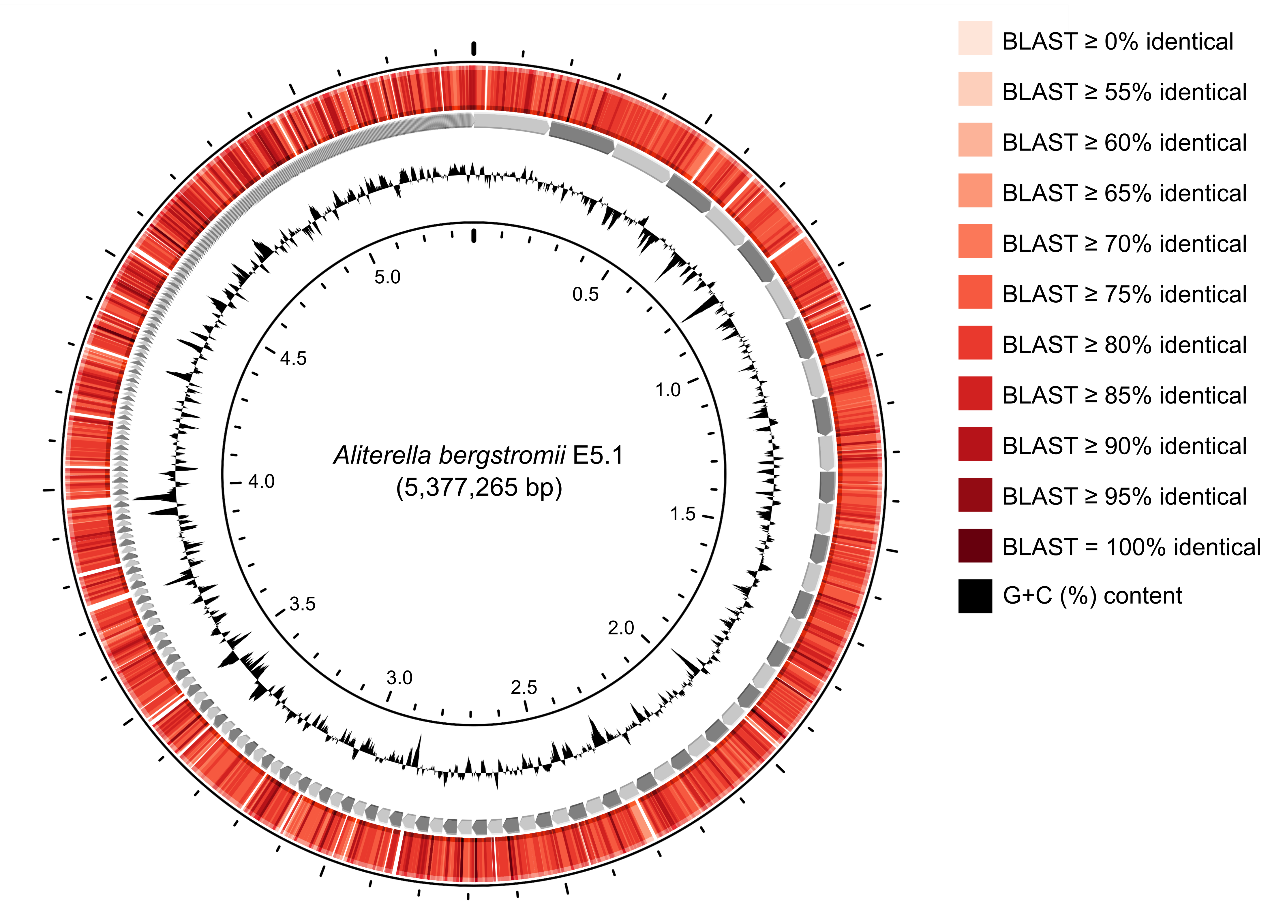
**

**Supplementary Figure 4.** Dot plot showing a genome-wide comparison between *Aliterella bergstromii* E5.1 and the closest available reference strain *Aliterella* sp. RAGGC 92. The alignments are coloured according to their sequence similarity.

**
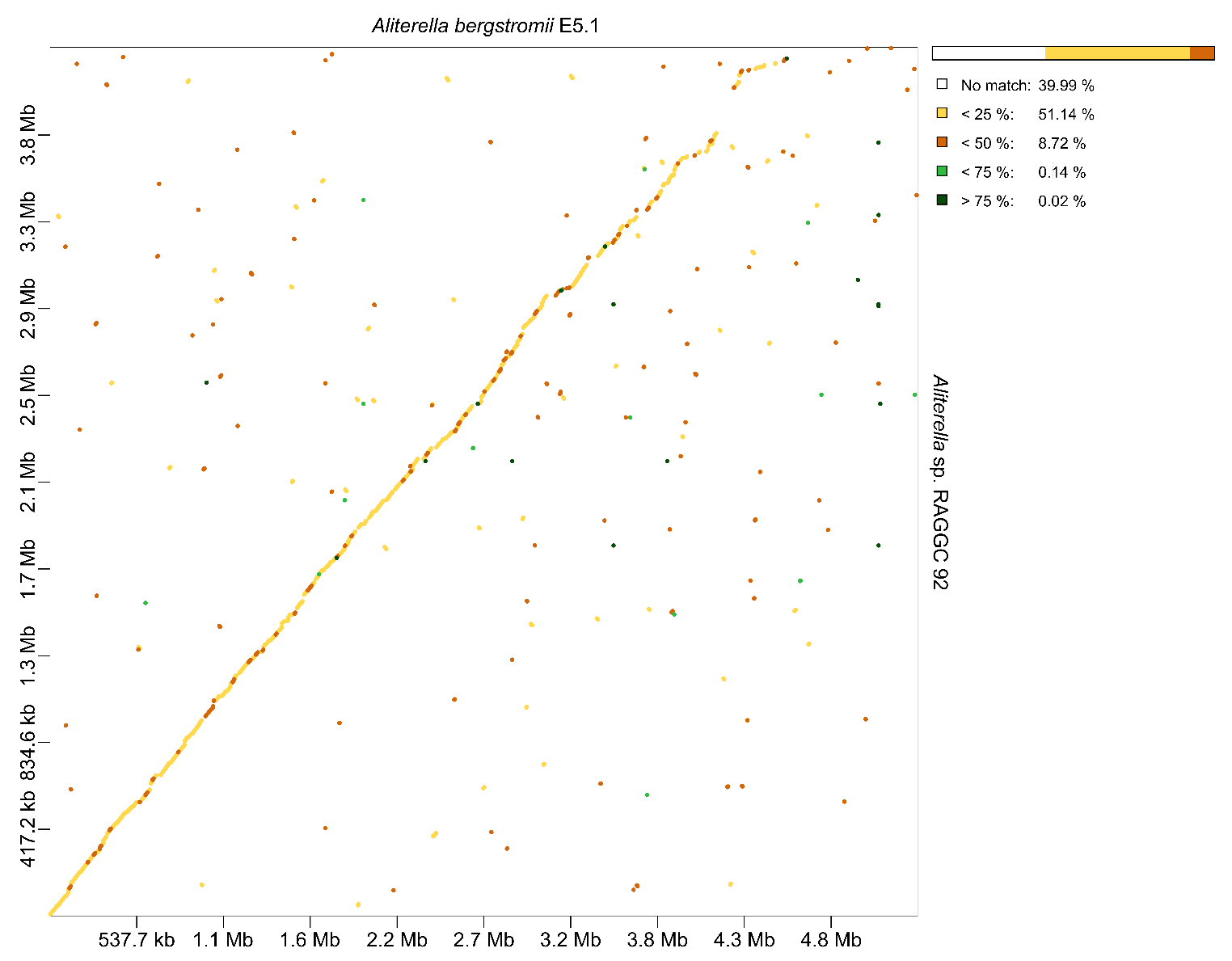
**
